# Profiles of developmental disorder and associations with gestational age

**DOI:** 10.1136/archdischild-2024-327962

**Published:** 2025-02-10

**Authors:** Katherine Jane Pettinger, Sarah Louise Blower, Elaine M Boyle, Catherine Elizabeth Hewitt, Lorna K Fraser

**Affiliations:** 1 Department of Health Sciences, University of York, York, UK; 2 Department of Population Health Sciences, University of Leicester, College of Life Sciences, Leicester, UK; 3 Cicely Saunders Institute, King's College London, London, UK

**Keywords:** Neonatology, Child Development, Epidemiology, Follow-Up Studies, Paediatrics

## Abstract

**Objective:**

This study aimed to examine profiles of co-occurrence of developmental disorders and their association with birth before full term.

**Design:**

Latent class analysis of cohort data with linked health data.

**Setting:**

Bradford, England.

**Patients:**

13 172 children were included in the analysis.

**Outcome measures:**

Developmental disorder in medical records.

**Methods:**

Data were censored at each child’s 12th birthday. The latent class analysis identified patterns of developmental disorders. Multinomial logistic regression explored the association with gestational age while adjusting for clinical and socio-factors.

**Results:**

The majority (12,536) had a low risk of developmental disorders; this group was named ‘typical development’. The remaining children were classified into three groups: ‘educational difficulties’ (347 children); ‘social, emotional, behavioural and communication difficulties’ (189 children) and ‘early developmental impairment, with physical and intellectual disabilities’ (100 children).

Compared with ‘typical development’, very preterm birth was associated with an increased likelihood of being in the ‘early developmental impairment, with physical and intellectual disabilities’ group, adjusted relative risk ratio (aRRR): 9.22 (95% CI 4.58 to 18.55). Birth before full term was associated with increased likelihood of being in the ‘educational difficulties’ group; risk was highest <34 weeks (aRRR: 2.64 (95% CI 1.44 to 4.83)) but persisted up to 37–38 weeks: aRRR: 1.41 (95% CI 1.10 to 1.81). There was no association between gestational age and the ‘social, emotional, behavioural and communication difficulties’’ group.

**Conclusion:**

Four distinct profiles of developmental disorders were identified; gestational age was associated with two of these. Understanding which disorders children are most at risk of and how these co-occur can help provide accurate information to families and contribute to prompt diagnosis.

**WHAT IS ALREADY KNOWN ON THIS TOPIC**

- Birth before full term is associated with increased risk and prevalence of developmental disorders.

**WHAT THIS STUDY ADDS**

- Latent class analysis can be used to identify four distinct profiles of developmental disorder: two of these were associated with gestational age. Children born late preterm (34–36 weeks) and early term (37–38 weeks) are at particular risk of educational difficulties, more so than physical disabilities.

**HOW THIS STUDY MIGHT AFFECT RESEARCH, PRACTICE OR POLICY**

- This highlights the risk of late preterm and early term birth and the need for improved integration of health and education services; education professionals may be able to direct children to specialised services where necessary.

## Introduction

 Children born before full term, including those born late preterm and early term, are at risk of developmental disorders.^1^ Existing research on the effects of preterm birth either uses broad outcome measures, such as educational achievement or special educational needs,^2 3^ or focuses on a single diagnosis, such as autism spectrum disorder (ASD),^4^ which neglects the reality that developmental disorders and their symptoms overlap and co-occur.^5 6^ It is important to further investigate the co-occurrence of developmental disorders and how these are associated with gestational age; anticipating challenges children are likely to face could assist prompt identification of needs, improving health and well-being.^7^ Children’s health and development may be affected differently by particular combinations of disorders. Categorising developmental disorders can help clinicians and educational professionals gain a clearer picture of children’s and families’ experiences beyond a single diagnosis.

Latent class analysis (LCA) uses observed data to reveal hidden (latent) groups or ‘classes’ within populations, which are distinct from one another but within which there is homogeneity.^8^ LCA has been used to explore profiles of developmental disorders, mainly using cohorts of children under investigation or treatment for a particular disorder, for example, attention deficit hyperactivity disorder (ADHD) or developmental coordination disorder.^9 10^ LCA has also been used to explore outcomes of children born preterm.^11^ Johnson *et al* identified three classes of development within their moderate-late preterm group: ‘optimal development’ and ‘non-optimal development’ with a high risk for behavioural problems and delayed social-emotional competence, and a third class with features similar to the very preterm phenotype. The study was limited by its reliance on parent-reported measures and short follow-up (2 years); problematic since developmental disorders are often diagnosed later (eg, ASD at 3.5 years and ADHD at 7 years^12 13^). Following children up into school age would allow a more thorough assessment of a range of outcomes. The aim of this study was to carry out an LCA to identify profiles of developmental disorders up to age 12 within routine health data and to explore whether gestational age at birth is associated with these profiles.

## Methods

Born in Bradford (BiB) was established to investigate the city’s high levels of child mortality and morbidity. Between 2007 and 2010, 12 453 pregnant women were recruited to the cohort, which is broadly reflective of the city’s maternal population.^14 15^ Ethical approval was granted by the Bradford Research Ethics Committee (Ref 07 /H1302/112).

For inclusion in this study, children needed gestational age data (maternity records) and primary care data; existing data linkage to external sources had been carried out by the BiB data team; routine health data linkage based on National Health Service (NHS) number, surname, gender and date of birth was available for 98% of cohort participants.^14^ Multiple births higher order than twins were excluded to avoid deductive disclosure. The dates of data extraction were 02 February 2023 and 24 May 2023 for primary and secondary care, respectively.

### Measures

The key exposure variable was gestational age in completed weeks.

The developmental problems and disorders listed in the National Institute for Health and Care Excellence (NICE) guideline for developmental follow-up of children born preterm^16^ were included:

1. Cerebral palsy.
2. Motor function problems
3. Intellectual/learning disability.
4. Special educational needs and low educational attainment (‘education problems’).
5. Executive function problems.
6. Speech, language, and communication problems
7. Hyperactivity, impulsivity and inattention, including ADHD.
8. ASD.
9. Emotional and behavioural problems.
10. Oro-motor feeding problems.
11. Visual impairment.
12. Hearing impairment.
13. Sleep apnoea.
14. Developmental delay (early developmental impairment).

Despite their inclusion in the NICE guideline, some conditions listed (eg, sleep apnoea) are not usually considered developmental disorders but were included here for completion. The guideline’s definition and list of developmental disorders were used, as it is a widely recognised evidence-based document. The broad range of conditions reflects the reality that developmental disorders often overlap and co-occur.^517^,^19^

### Case ascertainment

Clinical codes are entered into children’s records as part of their routine healthcare. Codes corresponding to the signs or symptoms of a developmental disorder could be recorded in a child’s record when they present to primary care or during an inpatient stay. In Bradford, developmental disorders are usually diagnosed in the child development centre, then communicated to the general practitioner, at which point the diagnosis is entered into the child’s primary care record.^20^

Clinical code lists corresponding to signs, symptoms or diagnosis of developmental disorders were developed using published lists (see online supplemental eTables 1–14). Participants’ medical records (primary and secondary care) were searched by the BiB data team to identify every code occurrence until the data extraction.

### Covariates

The selection of covariates was guided by the figurative representation (online supplemental eFigure 1). Covariates included sex (male/female), small for gestational age (SGA) (birth weight <10th centile, yes/no), multiplicity (singleton/twin), socioeconomic position produced from a previous study using 19 variables (including maternal education and receipt of means-tested benefits) measured during pregnancy^21^ (least deprived and most educated, employed not materially deprived, employed no access to money, receiving benefits but coping and most deprived), maternal age at delivery (<21, 21–25, 26–30, 31–35 and >35 years), ethnicity (white, mixed, Asian, black and other) and maternal smoking during pregnancy (yes/no).

### Data derivation

To preserve anonymity, only the birth month and year were provided by BiB. To calculate approximate age, days of birth were set to the first of each month. Data were censored at the child’s (approximated) 12th birthday.

### Statistical analysis

Data analysis was performed from February 2023 to May 2024 as follows:

1. LCA.
2. Class membership described.
3. Modelling the association between latent classes and gestational age.

Analyses were undertaken in Stata/SE software V.18 and R V.4.3.2.^22 23^ LCA was undertaken using the poLCA package in R.^24^

### Latent class analysis

A one-class model was used as a baseline, followed by an exploration of 2–5 classes.^25^

The optimal solution was chosen based on fit statistics and classification quality. Fit was evaluated using the Akaike information criterion, Bayesian information criterion (BIC), consistent Akaike’s and sample-size adjusted BIC, with lower values indicating a better fit and BIC considered most reliable.^25^ Classification quality was evaluated using relative entropy and average posterior probability, with values >0.8 and >0.7 considered acceptable, respectively.^26^ When the optimal number of classes was chosen, profiles were described. Children were classified into groups according to maximum posterior probability.^27^

### Adjusted multinomial regression

The association between gestational age and class membership was explored using multinomial regression with the largest class as the reference while adjusting for covariates. Gestational age was presented in categories: <34 weeks (‘very/moderate preterm’), 34–36 weeks (‘late preterm’), 37–38 weeks (‘early term’), 39–41 weeks (‘full term’) and >41 weeks (‘post-term’). Missing data were imputed using multiple imputations by chained equations.^28^

### Results

There were 13 172 children in the analysis cohort (online supplemental eFigure 2). There were 324 children who were withdrawn and 53 who died; these data were included up until the point of death/withdrawal.

The four-class model was chosen as it had the lowest BIC and high classification quality (relative entropy: 0.95 and average posterior probability: 0.75–0.99) (online supplemental eTable 15, online supplemental eFigure 3). Distributions of predicted probabilities for each participant across the four latent classes, divided by assigned and non-assigned groups, are shown in online supplemental eFigure 4.

**Figure 1:**
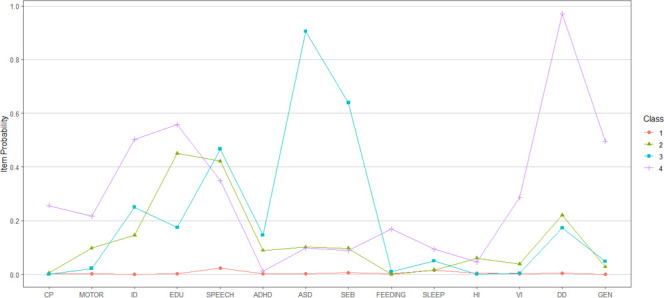
Final latent class analysis model for developmental disorders (n=13 172). ADHD, hyperactivity, impulsivity and inattention, including attention deficit hyperactivity disorder; ASD, autism spectrum disorder; CP, cerebral palsy; DD, developmental delay; EDU, education problem; FEEDING, feeding problems; GEN, general developmental disorder; HI, hearing impairment; ID, intellectual disability; MOTOR, non-CP motor problem; SEB, social, emotional, and behavioural problems; SLEEP, sleep apnoea; SPEECH, speech, language and communication disorders; VI, visual impairment. NB, there were no codes in participants’ records corresponding to executive function problems.

The profiles of developmental disorders differed in terms of the probability of each disorder (figure 1 and table 1) and clinical and sociodemographic characteristics (table 2).

**Table 1:** Results for the latent class analysis (four-class solution)

	Full sample	Group 1: ‘typical development’	Group 2: ‘educational difficulties’	Group 3: ‘social, emotional, behavioural and communication difficulties’	Group 4: ‘early developmental impairment, with physical and intellectual disabilities’
Number of children	13 172	12 536	347	189	100
Average posterior probabilities	N/A	0.99	0.75	0.89	0.87
Cerebral palsy	0.33%	0.11%	0.59%	0%	25.57%
Motor function problems	0.69%	0.17%	9.80%	2.20%	21.61%
Intellectual disability	1.28%	0%	14.50%	24.98%	50.22%
Education problem	2.46%	0.29%	45.06%	17.44%	55.74%
Speech, language and communication disorders	4.55%	2.29%	42.11%	46.70%	34.84%
Attention deficit hyperactivity disorder	0.74%	0.23%	8.88%	14.56%	1.09%
Autism spectrum disorder	2.08%	0.23%	10.15%	90.50%	9.80%
Social, emotional and behavioural problems	2.04%	0.67%	9.69%	63.95%	8.75%
Feeding problems	0.38%	0.24%	0%	0.95%	16.91%
Sleep apnoea	1.66%	1.53%	1.61%	5.01%	9.33%
Hearing impairment	0.59%	0.38%	6.04%	0%	4.64%
Visual impairment	0.62%	0.27%	3.84%	0.37%	28.52%
Developmental delay	2.08%	0.31%	22.07%	17.26%	97.09%
General/unclassifiable	0.64%	0.06%	2.74%	4.80%	49.56%

Total sample: 13 172. Probability of each developmental disorder by latent class. See also figure 1.

**Table 2:** Distribution of cohort characteristics by class membership

	Group 1: ‘typical development’	Group 2: ‘educational difficulties’	Group 3: ‘social, emotional, behavioural and communication difficulties’	Group 4: ‘early developmental impairment, with physical and intellectual disabilities’	Total
Gestational age					
<34 weeks	190	12	*	*	214
	1.5%	3.5%			1.6%
34–36 weeks	569	22	*	*	606
	4.5%	6.3%			4.6%
37–38 weeks	2786	94	47	23	2950
	22.2%	27.1%	24.9%	23%	22.4%
39–41 weeks	8823	212	128	59	9222
	70.4%	61.1%	67.7%	59%	70%
>41 weeks	168	*	*	*	180
	1.3%	*	*	*	1.4%
Total	12 536	347	189	100	13 172
	100%	100%	100%	100%	100%
Sex					
Male	6357	223	159	62	6801
	50.7%	64.3%	84.1%	62%	51.6%
Female	6179	124	30	38	6371
	49.3%	35.7%	15.9%	38%	48.4%
Total	12 536	347	189	100	13 172
	100%	100%	100%	100%	100%
Multiplicity					
Singleton	12 247	335	*	*	12 867
	97.7%	96.5%			97.7%
Twin	289	12	*	*	305
	2.3%	3.5%			2.3%
Total	12 536	347	189	100	13 172
	100%	100%	100%	100%	100%
SGA					
Not SGA	10 557	268	165	77	11 067
	86.2%	80%	89.2%	77%	86%
SGA	1685	67	20	23	1795
	13.8%	20%	10.8%	23%	14%
Total	12 242	335	185	100	12 862
	100%	100%	100%	100%	100%
Maternal age at delivery (years)					
<21	1268	31	24	11	1334
	10.1%	8.9%	12.7%	11%	10.1%
21–25	3618	100	51	29	3798
	28.90%	28.8%	27%	29%	28.8%
26–30	4000	110	60	31	4201
	31.9%	31.7%	31.8%	31%	31.9%
31–35	2436	63	27	19	2545
	19.4%	18.2%	14.3%	19%	19.3%
>35	1214	43	27	10	1294
	9.7%	12.4%	14.3%	10%	9.8%
Total	12 536	347	189	100	13 172
	100%	100%	100%	100%	100%
Smoking during pregnancy					
Smoking	1693	49	35	*	1782
	16.4%	16.7%	22.3%		16.4%
No smoking	8659	244	122	*	9103
	83.7%	83.3%	77.7%		83.6%
Total	10 352	293	157	83	10 885
	100%	100%	100%	100%	100%
Ethnicity					
White	4749	136	94	22	5001
	38.1%	39.2%	49.7%	22%	38.2%
Mixed	671	10	18	*	704
	5.4%	2.9%	9.5%		5.40%
Asian	6633	194	71	72	6970
	53.2%	55.9%	37.6%	72%	53.2%
Black	234	*	*	*	245
	1.90%				1.9%
Other	176	*	*	*	179
	1.4%				1.4%
Total	12 463	347	189	100	13 099
	100%	100%	100%	100%	100%
Socioeconomic position					
Least deprived, most educated	2016	37	33	*	2096
	19.6%	12.7%	21%		19.3%
Employed not materially deprived	2070	47	37	*	2160
	20.1%	16.2%	23.6%		19.9%
Employed no access to money	1574	34	30	18	1656
	15.3%	11.7%	19.1%	22%	15.3%
Benefits but coping	3023	112	31	35	3201
	29.3%	38.5%	19.8%	42.7%	29.5%
Most deprived	1630	61	26	13	1730
	15.8%	21%	16.6%	15.9%	16%
Total	10 313	291	157	82	10 843
	100	100	100	100	100

* Data supressed in ≥2 cells if fewer than 10 children in one or more cells, to avoid deductive disclosure.

SGA, small for gestational age.

*Group 1: ‘typical development’*—the largest group, 12 536 children. These children had a low chance of any developmental disorders.

*Group 2: ‘educational difficulties’*—347 children. Children often had educational problems (45%), for example, dyslexia/dyscalculia (online supplemental eTable 4) but a relatively low probability (14.5%) of intellectual disability. This group had the highest (6% vs 0.6% in the full sample) probability of hearing impairment.

*Group 3: ‘social, emotional, behavioural and communication difficulties’*—189 children. There was a high (90.5%) probability of ASD and social, emotional and behavioural problems (64%). This group had the highest likelihood of ADHD (14.6%) and speech, language and communication disorders (46.7%).

*Group 4: ‘early developmental impairment, with physical and intellectual disabilities’*—100 children. This group was the most likely to have CP (25.6%), intellectual disability (50.2%), motor function problems (21.6%), feeding problems (16.9%) and sleep apnoea (9.3%). There was a 97.1% probability of developmental delay/early developmental impairment.

The distribution of covariates differed by latent class (table 2). Compared with ‘typical development’, there was a higher proportion of boys in all other groups, especially ‘social, emotional, behavioural and communication difficulties’ (84.1% male). 20% of ‘educational difficulties’ and 23% of ‘early developmental impairment, with physical and intellectual disabilities’ were SGA, compared with 14% of the whole cohort.

Details of missing data and diagnostics of the imputation are shown in the supplement, online supplemental eTable 16.

**Figure 2:**
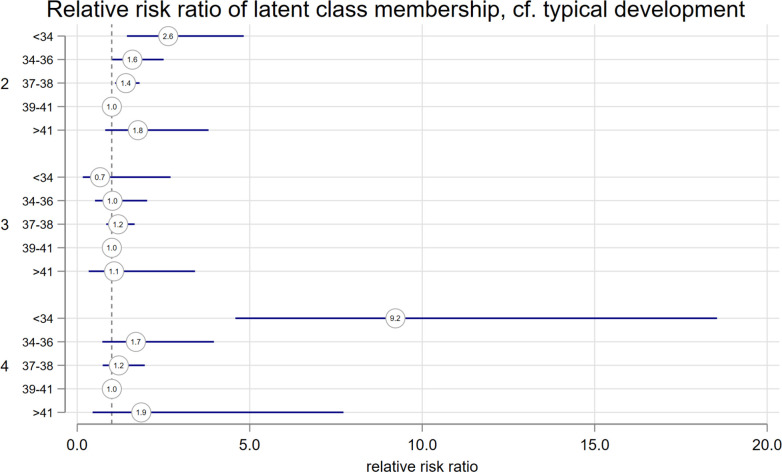
Coefficient plot shows the adjusted relative risk ratio of latent class membership according to gestational age, compared with class 1 ‘typical development’. Imputed dataset. Adjusted for child’s sex, small for gestational age, maternal age, maternal smoking, socioeconomic position and ethnicity. Multiplicity was omitted from regression due to collinearity.

There was increased risk of membership of Group 2 ‘educational difficulties’, compared with ‘typical development’ in all groups less than full term; adjusted relative risk ratios (aRRR) <34 weeks: 2.64 (95% CI 1.44 to 4.83); 34–36 weeks: 1.60 (95% CI 1.02 to 2.50) and 37–38 weeks: 1.41 (95% CI 1.10 to 1.81) (figure 2, table 3). There was an increased aRRR of membership in the ‘early developmental impairment, with physical and intellectual disabilities’ group, compared with ‘typical development’ in the <34-week group; 9.22 (95% CI 4.58 to 18.55). There was no association between gestational age and membership of the ‘social, emotional, behavioural and communication difficulties’ group.

**Table 3:** Multivariable multinomial logistic regression using imputed dataset

	RRR	95% CI		P value	RRR	95% CI		P value	RRR	95% CI		P value
	Group 2: ‘educational difficulties’, n=347				Group 3: ‘social, emotional, behavioural and communication difficulties’, n=189				Group 4: ‘early developmental impairment, with physical and intellectual disabilities’, n=100			
Gestational age categories (weeks)												
<34	2.64	1.44	4.83	0.002	0.66	0.16	2.71	0.6	9.22	4.58	18.55	<0.0001
34–36	1.60	1.02	2.5	0.04	1.02	0.51	2.03	0.96	1.70	0.73	3.96	0.2
37–38	1.41	1.10	1.81	0.007	1.18	0.84	1.66	0.3	1.20	0.74	1.96	0.5
39–41	1.00 (ref)				1.00 (ref)				1.00 (ref)			
>41	1.76	0.81	3.81	0.2	1.07	0.33	3.41	0.9	1.85	0.45	7.72	0.4
Child’s sex												
Female	1.00 (ref)				1.00 (ref)				1.00 (ref)			
Male	1.78	1.42	2.22	<0.0001	5.09	3.44	7.54	<0.0001	1.63	1.09	2.45	0.02
SGA												
SGA	1.6	1.21	2.11	0.001	0.86	0.53	1.38	0.5	1.76	1.09	2.85	0.02
Not SGA	1.00 (ref)				1.00 (ref)				1.00 (ref)			
Maternal age (years)												
<21	0.8	0.5	1.25	0.3	1.55	0.87	2.79	0.1	1.36	0.62	2.96	0.4
21–25	0.99	0.72	1.37	0.96	1.33	0.83	2.16	0.2	1.07	0.59	1.94	0.8
26–30	1.04	0.76	1.43	0.8	1.39	0.88	2.2	0.2	1.02	0.57	1.82	1
31–35	1.00 (ref)				1.00 (ref)				1.00 (ref)			
>35	1.32	0.89	1.97	0.2	1.97	1.14	3.38	0.01	1.08	0.5	2.36	0.8
Maternal smoking in pregnancy (any)												
No smoking	1.00 (ref)				1.00 (ref)				1.00 (ref)			
Smoking	0.91	0.62	1.33	0.6	1.15	0.74	1.78	0.5	0.32	0.12	0.85	0.02
Socioeconomic position												
Least deprived and most educated	1.00 (ref)				1.00 (ref)				1.00 (ref)			
Employed, not materially deprived	1.21	0.77	1.88	0.4	0.88	0.52	1.47	0.6	0.73	0.27	1.99	0.5
Employed, no access to money	1.28	0.8	2.04	0.3	0.99	0.59	1.66	0.96	2.36	1.11	5.00	0.03
Benefits not materially deprived	2.08	1.42	3.04	<0.0001	0.64	0.39	1.06	0.09	2.12	1.04	4.31	0.04
Most economically deprived	2.2	1.44	3.37	<0.0001	0.84	0.48	1.48	0.5	2.12	0.95	4.72	0.07
Ethnicity												
White	1.00 (ref)				1.00 (ref)				1.00 (ref)			
Mixed	0.48	0.25	0.93	0.03	1.37	0.82	2.29	0.2	1.43	0.53	3.82	0.5
Asian	0.84	0.64	1.1	0.2	0.62	0.43	0.89	0.009	1.51	0.88	2.6	0.1
Black	0.84	0.36	1.94	0.7	0.9	0.32	2.51	0.8	0.67	0.09	5.09	0.7
Other	0.21	0.03	1.51	0.1	0.6	0.15	2.5	0.5	0	0	.	0.99

n=13 172.

Class 4: ‘typical development’ is reference class.

RRR, relative risk ratio; SGA, small for gestational age.

## Discussion

This latent class analysis has revealed four profiles of the coexistence of developmental disorders. The majority of children have ‘typical development’ and are unlikely to have any developmental disorders. Two classes of developmental disorders were associated with birth before full term. There was an increased aRRR of ‘educational difficulties’ for all groups born before full term. The effect of gestational age was larger in the ‘early developmental impairment, with physical and intellectual disabilities’ group but only statistically significant in the very/moderate preterm group. In short, children born late preterm and early term may need help in school but are unlikely to have physical and intellectual disabilities as a result of immaturity.

Babies born very preterm are at risk of specific ‘preterm pathologies’, such as intraventricular haemorrhage and retinopathy of prematurity; these conditions are rare beyond 34 weeks gestation, and yet these children have been shown to be at increased risk of developmental disorders in general.^1^ This study has shown that late preterm and early term children are at increased risk of a different profile of disorders compared with very/moderately preterm children. This is likely reflective of the different pathophysiological processes occurring in births before full term; even in the absence of structural brain anomalies, the evolution of typical neural connections and brain maturation may be disrupted if born late preterm or early term.^29 30^

Group 3 ‘social, emotional, behavioural and communication difficulties’ weech impairment, ias not associated with gestational age. This was surprising, as previous studies have shown that birth before full term is associated with an increased likelihood of ASD and ADHD.^1^ It is feasible that these conditions are not as strongly associated with gestational age as other developmental disorders. Parenting, as well as other environmental factors, is known to influence children’s social, emotional and behavioural development.^31^ Genetics could play a role; ASD and ADHD have a heritability of 50% and 74%, respectively.^32 33^ Boys are known to be more likely than girls to be diagnosed with ASD and ADHD,^34^ as is demonstrated here; in the ‘typical development’ group, 50.7% were male, whereas in the ‘social, emotional, behavioural and communication difficulties’ group, 84.1% were male. Groups 2 and 4 were 64.3% and 62.0% male, respectively; while male sex predisposes to all developmental disorders, the association is particularly clear in Group 3 and may eclipse the importance of gestational immaturity. This cohort also contains a high proportion of Asian children, who may be less likely to receive an ASD diagnosis.^35^

While there was a much higher probability of speech, language and communication disorders in the non-typical development groups, for example, 63.95% in Group 3 ‘social, emotional, behavioural and communication difficulties’ and 9.7% in Group 2 ‘educational difficulties’, since there were 12 536 children in the ‘typical development’ group, it is likely that the majority of children presenting with a speech, language and communication disorder go on to have no other developmental disorders. Clinicians need to discern between types of speech, language and communication disorder, in particular lack of communicative intent, as distinct from an isolated speech impairment, in order to discern between children who will go on to develop typically, compared with those who may have more complex journeys ahead.

Johnson *et al* assessed a moderate-to-late preterm and a term cohort at 2 years. They identified three classes of development in moderate-to-late preterm children: ‘optimal development’, ‘non-optimal development’ (behavioural problems, delayed social-emotional competence) and ‘very preterm development’ (cognitive and language delays).^11^ This analysis was different, using a single cohort and multinomial regression to explore the relationship with gestational age. Camerota *et al* used latent profile analysis to explore 2-year outcomes of children born very preterm. They identified four developmental profiles and found that approximately 85% of children had a low probability of developmental delay or behavioural problems.^36^ In our <34-week group, we found 88.9% of the children were categorised in the ‘typical development’ profile; however, this only included 21 children <28 weeks. Twilhaar *et al* also explored outcomes among very preterm children (n=1977) using latent profile analysis, at 5 years. They identified four profiles: no deficit in any domain (45%), motor and cognitive deficits without behavioural/psychosocial deficits (31%), primarily behavioural and psychosocial deficits (16%) and deficits in multiple domains (8%).^37^

In England, approximately 3–4% of children have a developmental disorder. There are 636 children in this cohort (4.8%) who fall into one of the non-typical development classes; the incidence of developmental disorders in this cohort may be higher than average. Considering established links between developmental disorders and poverty and deprivation in Bradford, this is unsurprising.^15 38^

### Implications for practice

Clinical and sociodemographic factors can be used to help predict children’s developmental profiles and assess the risk of developmental disorders. This could facilitate earlier diagnosis and intervention. However, the majority of children in the cohort have typical development, which is important when counselling parents to balance this against preparing parents for potential problems their children are at risk of.

Understanding how disorders co-occur can help clinicians anticipate a child’s needs. For example, in Group 4, there was a 50.2% probability of intellectual disability, a 25.6% probability of CP and a 28.5% probability of visual impairment. For children born <34 weeks, the aRRR for membership in Group 4 was 9.22 (95% CI 4.58 to 18.55); moderately/very preterm children should be monitored for these disorders and might benefit from visual screening and physiotherapy assessment for motor disorders.

Parents should be encouraged to inform schools if their child was born before full term, since membership of Group 2 ‘educational difficulties’ was associated with all groups of children born before full term and was the largest group of children with atypical development. This is in keeping with research showing an increased prevalence of special educational needs and low educational attainment among children born before full term.^1^ Late preterm and early term children are more likely to experience specific problems such as dyscalculia and dyslexia rather than intellectual disability, which would require different forms of educational support, for example, assistive technology to improve accessibility.^39^ There has been very limited research into the association between dyslexia specifically and gestational age, although a recent UK study has demonstrated a link.^40^

### Limitations

This study relies on the accuracy of coding within the participants’ medical records. Codes were entered into the children’s medical records as part of their healthcare, that is, not for research purposes; no specific testing for this study was carried out; the absence of a clinical code does not guarantee the absence of a disorder. While the case ascertainment strategy was broad, to account for unusual coding practices as well as differences in how ethnic groups access healthcare,^41^ it is possible that codes may have been omitted or entered incorrectly in children’s records.

The BiB cohort is largely biethnic (online supplemental eTable 17); aside from the Asian and white groups, the remaining ethnic groups were too small to investigate. However, this is still a valuable advancement, given how little research has been carried out previously into the outcomes of children from minoritised ethnic groups born before full term.^1^ Future research should endeavour to include a wider range of ethnicities.

There were very few extremely preterm children, partly due to the recruitment strategy (at 26–28 weeks), and overall, there were fewer preterm infants (6.3%) in the cohort compared with the national average (7.6%).^42^ While limited conclusions about children born <28 weeks can be drawn, there were sufficient preterm children to find statistically and clinically significant relationships between class membership for all categories of birth before full term and ‘educational difficulties’ and birth <34 weeks gestation and ‘early developmental impairment, with physical and intellectual disabilities’.

The study offers a snapshot of developmental disorders, which may not fully represent children’s developmental trajectories. BiB is continuing to follow this cohort; subsequent research exploring outcomes aged 16–18 may offer a more complete picture.

Three classes contained less than 5% of the analysis cohort. Previously, this was thought to be problematic; however, recent LCA research suggests that whether the class ‘makes conceptual sense’ is more important.^25^

## Conclusion

Four profiles were identified, which varied in type and combinations of developmental disorders. Birth before full term increased the likelihood of being in the ‘educational difficulties’ or ‘early developmental impairment, with physical and intellectual disabilities’ groups, compared with typical development. Understanding which disorders children are at risk of can help provide individualised information to families and contribute to prompt diagnosis.

## Supplementary material

10.1136/archdischild-2024-327962 online supplemental file 1

## Data Availability

Data may be obtained from a third party and are not publicly available.
