# Supplementary material for: Profiles of developmental disorder and associations with gestational age
Source: Arch Dis Child. 2025 Feb 10;110(8):e327962. doi: 10.1136/archdischild-2024-327962 (PMC12320604; doi:10.1136/archdischild-2024-327962)
Supplement: online supplemental file 1 [file archdischild-110-8-s001.pdf]

Supplemental Online Content

eFigure 1 - Figurative representation (conceptual model) showing the associations between exposure (birth before full term) and outcome (developmental disorder)

eFigure 2 – Sample selection, study flow chart

eTable 1 – Codes grouped as cerebral palsy

eTable 2 – Codes grouped as non-cerebral palsy motor problems

eTable 3 – Codes grouped as intellectual disability (cognitive impairment). NB, it the UK, the term learning disability is synonymous with intellectual disability

eTable 4 – Codes grouped as educational problems

eTable 5 – Codes grouped as speech, language, and communication problems

eTable 6 – Codes grouped as Attention, impulsivity, and hyperactivity (including ADHD)

eTable 7 – Codes grouped as Autism Spectrum Disorder

eTable 8 – Codes grouped as Social, Emotional, and Behavioural problems

eTable 9 – Codes grouped as Feeding problems

eTable 10 – Codes grouped as Sleep problems

eTable 11 – Codes grouped as Visual impairment

eTable 12 – Codes grouped as Hearing impairment

eTable 13 – Codes grouped as Developmental delay (also known as 'early developmental impairment")

eTable 14 – Codes unclassified / 'general' developmental disorder

eTable 15 - Model fit indices for Latent Class analysis models with 1 to 5 classes.

eFigure 3 - Elbow plot

eTable 16 – Imputation diagnostics

eTable 17 – Cohort description. Comparison of whole Born in Bradford cohort and, analysis cohort.

eFigure 4 – Distributions of predicted probabilities for each participant across four latent classes, divided by assigned and non-assigned groups

References

eFigure 1 - Figurative representation showing possible associations between exposure (birth before full term), outcome (developmental disorder) and co-variables
Orange box = independent variable; yellow box= dependent variable; grey boxes= possible mediating variables; blue box = direct effect of immaturity; green boxes = possible confounders; blue arrows = connection between covariate; orange arrows = covariates which may be influenced by birth before full term .
ROP: Retinopathy of Prematurity
IVH: Intraventricular Haemorrhage

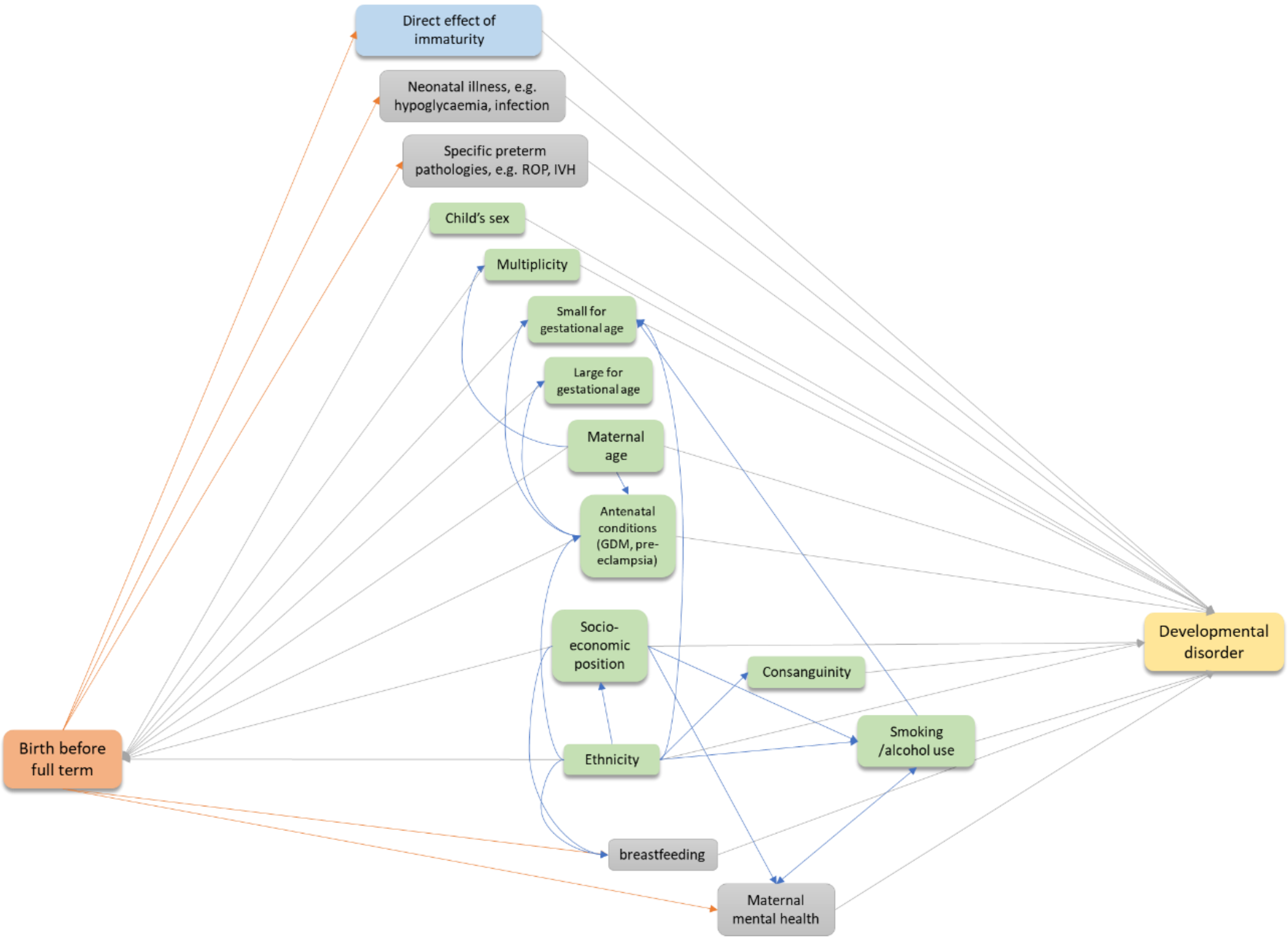

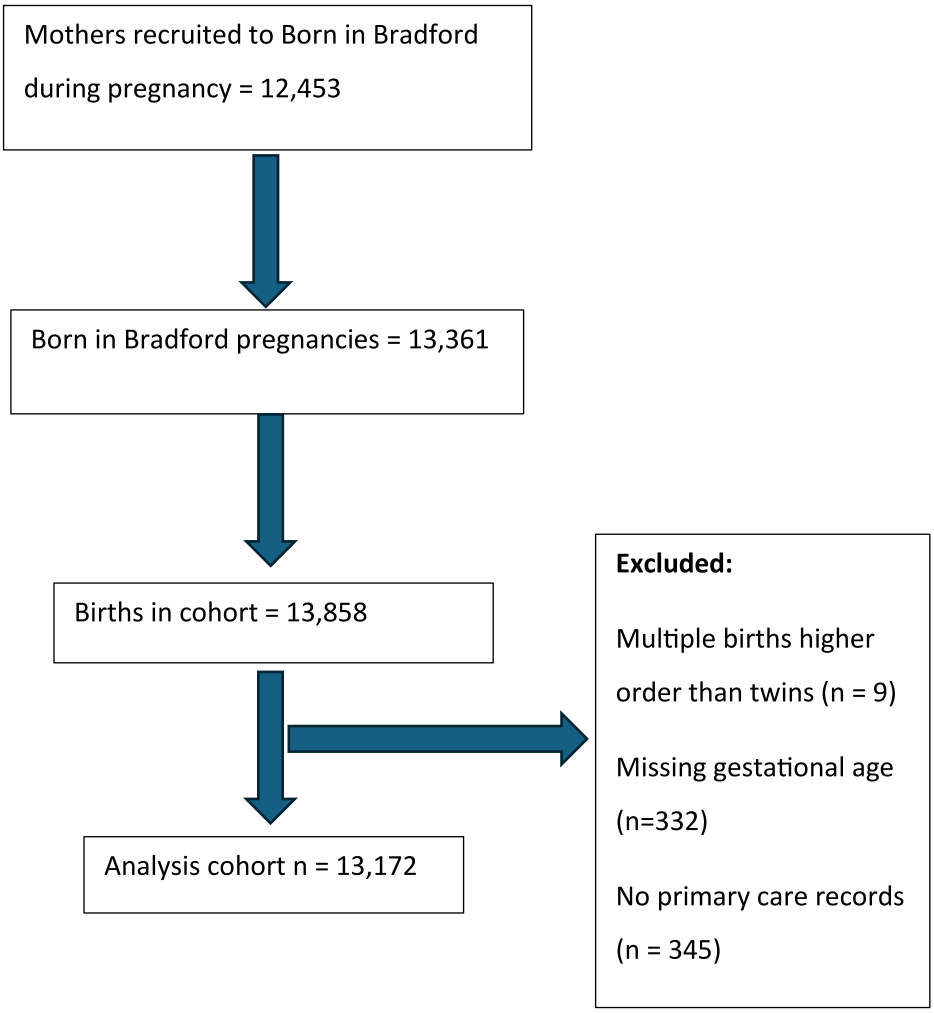

eFigure 2 – Sample selection, study flow chart

Clinical code lists

Clinical code lists corresponding to signs, symptoms, or diagnosis of developmental disorders were developed using published lists (see eTables 1 -14) [1-5] as well as by searching the Clinical Terminology Browser and NHS Digital's Classifications Browser.[6] The codes which were split into categories corresponding to each disorder based on existing published lists, in accordance with the hierarchical structure of the codes, and in discussion with paediatricians.

CTV3 = Clinical terms Version 3, used in primary care records. ICD-10, International Classification of Diseases – 10th edition, used in secondary care records.

eTable 1 – codes grouped as cerebral palsy

CTV3 Terms
[X]Other infantile cerebral palsy
Bilateral spastic cerebral palsy
Cerebral palsy
Cerebral palsy with spastic diplegia
Congenital diplegia NOS
Diplegia
Gross Motor Function Classification System for CP level II
Hemiplegia
Infantile cerebral palsy
Left hemiplegia
Monoplegia of upper limb
Right hemiplegia
Spastic cerebral palsy
Spastic diplegia
Spastic hemiplegia
Spastic hemiplegic cerebral palsy
Spastic quadriplegic cerebral palsy
ICD-10 term
Cerebral palsy, unspecified
Diplegia of upper limbs
Hemiplegia - unspecified
Hemiplegia, unspecified
Infantile cerebral palsy - unspecified
Infantile cerebral palsy, unspecified
Infantile hemiplegia
Monoplegia - unspecified
Monoplegia, unspecified
Other cerebral palsy
Other infantile cerebral palsy
Paralytic syndrome - unspecified
Spastic cerebral palsy
Spastic cerebral palsy

Spastic diplegia
Spastic diplegic cerebral palsy
Spastic hemiplegic cerebral palsy
Spastic tetraplegia
Tetraplegia, unspecified

eTable 2 – Codes grouped as non-cerebral palsy motor problems

CTV3 Terms
[D]Fine motor skills development delay
[D]Gross motor skills development delay
Apraxia
Ataxia
Cerebellar ataxia
Clumsiness
Clumsiness -motor delay
Coordination problem
DCD - developmental coordination disorder
Developmental disorder of motor function
Developmental verbal dyspraxia
Dyspraxia
Motor developmental delay
Not yet sitting
Not yet walking
O/E - clumsy
O/E - generally unsteady
Oculomotor apraxia
Poor coordination
Truncal ataxia
ICD-10 term
Ataxia - unspecified
Other and unspecified lack of coordination
Specific developmental disorder of motor function

eTable 3 – codes grouped as intellectual disability (cognitive impairment). NB, it the UK, the term learning disability is synonymous with intellectual disability.

CTV3 Terms
[V]Problems with learning
[X]Unsp mental retard with statement no or min impairm behav
[X]Unspecified mental retardation
Difficulty comprehending language
Learning disabilities annual health assessment
Learning disabilities health action plan completed
Learning disabilities health action plan declined

Learning disabilities health action plan offered
Learning disabilities health action plan reviewed
Learning disabilities health assessment
Learning disability
Learning disability health examination
Mild learning disability
Mild mental retardation, IQ in range 50-70
Moderate learning disability
On learning disability register
Profound learning disability
Seen in learning disabilities clinic
Severe learning disability
Significant learning disability
Specific learning disability
ICD-10 term
Mild mental retardation without mention of impairment behav
Mild mental retardation; Without mention of impairment of behaviour
Mild mental retard with statement no or min impairm behav
Mod mental retard without mention of impairment of behav
Profound mental retard without mention of impairm behav
Profound mental retardation; Without mention of impairment of behaviour
Severe mental retard without mention of impairment of behav
Severe mental retardation; Significant impairment of behaviour requiring attention or treatment
Severe mental retardation; Without mention of impairment of behaviour
Unspec mental retard without mention of impairment of behav
Unspec mental retardation
Unspecified mental retardation; Other impairments of behaviour
Unspecified mental retardation; Significant impairment of behaviour requiring attention or treatment
Unspecified mental retardation; Without mention of impairment of behaviour
Other and unspecified symptoms and signs involving cognitive functions and awareness
Oth/unsp sympt & signs involv cognitive funct & awareness

eTable 4 – Codes grouped as educational problems

CTV3 Terms
[X]Developmental disorder of scholastic skills, unspecified
Developmental disorder of scholastic skill
Difficulty reading
Difficulty writing
Dyscalculia
Dyslexia
Learning difficulties
Other specific learning difficulty
Unable to write
ICD-10 term

Developmental disorder of scholastic skills - unspecified
Developmental disorder of scholastic skills, unspecified
Other problems related to education and literacy
Specific reading disorder
Specific spelling disorder

eTable 5 – Codes grouped as speech, language, and communication problems

CTV3 Terms
(Develop disord: [language][speech]) or (articulatn defect)
[D]Communication skills development delay
[V]Problems with communication, including speech
[X]Developmental disorder of speech and language unspecified
[X]Receptive language disorder
Developmental expressive language disorder
Developmental language delay
Developmental language disorder
Developmental language impairment
Developmental speech articulation disorder
Developmental speech disorder
Disorder of speech and language development
Dysfluency
Expressive language delay
Expressive language disorder
Expressive language impairment
Language development disorder
Language impairment
Not yet speaking
O/E - speech delay
Phonological delay
Phonological disorder
Receptive language delay
Receptive language impairment
Restricted language development
Sociolinguistic difficulties
Specific language impairment
Speech and language disorder
Speech delay
Speech impairment
Speech or language developmental disorder NOS
Word finding difficulty
ICD-10 term
Developmental disorder of speech & language - unspecified
Developmental disorder of speech and language, unspecified
Specific speech articulation disorder
Stuttering [stammering]

eTable 6 – Codes grouped as Attention, impulsivity, and hyperactivity (including ADHD)

CTV3 Terms
[X]Hyperkinetic disorder, unspecified
Attention deficit hyperactivity disorder
Attention deficit without hyperactivity
Child attention deficit disorder
Childhood hyperkinetic syndrome
Poor concentration
Reduced concentration
Reduced concentration span
Unable to concentrate
ICD-10 term
Disturbance of activity & attention
Disturbance of activity and attention
Hyperkinetic disorder, unspecified

eTable 7 – Codes grouped as Autism Spectrum Disorder

CTV3 Terms
[X]Other childhood disintegrative disorder
Active infantile autism
Atypical autism
Autistic spectrum disorder
Autistic spectrum disorder - Autism checklist
Autistic spectrum disorder - Gluten-casein intolerance checklist
Childhood autism
ICD-10 term
Atypical autism
Childhood autism
Pervasive developmental disorder, unspecified

eTable 8 – Codes grouped as Social, Emotional, and Behavioural problems

CTV3 Terms
[D]Social skills development delay
[V]Mental and behavioural problems
[V]Other behavioural problems
[V]Unspecified mental or behavioural problem
Adolescent - emotional problem
Aggressive outburst
Behavioural and emotional disorder with onset in childhood
Behavioural, emotional, and social difficulties
Childhood disorder of conduct and emotion
Childhood phobic anxiety disorder

Childhood social anxiety disorder
Disturbance anxiety and fearfulness childhood/adolescent NOS
Emotional behavioural difficulties
Pica of infancy and childhood
Psychogenic feeding disorder of infancy and childhood
ICD-10 term
Childhood disorder of social functioning, unspecified
Combined vocal and multiple motor tic disorder [de la Tourette]
Conduct disorder - unspecified
Conduct disorder, unspecified
Educational maladjustment and discord with teachers and classmates
Elective mutism
Irritability & anger
Irritability and anger
Other childhood emotional disorders
Other conduct disorders
Other symptoms & signs involving emotional state
Other symptoms and signs involving emotional state
Other tic disorders
Physical violence
Pica of infancy and childhood
Restlessness & agitation
Restlessness and agitation
Tic disorder - unspecified
Tic disorder, unspecified

eTable 9 – Codes grouped as Feeding problems

CTV3 Terms
[D]Feeding difficulties NOS
[D]Feeding problem in infant
Feeding problem in child
Feeding problem NOS
Feeding problem symptom
ICD-10 term
Feeding difficulties & mismanagement
Feeding difficulties and mismanagement
Feeding disorder of infancy and childhood

eTable 10 – Codes grouped as Sleep problems

CTV3 Terms
[D]Sleep apnoea syndrome
Obstructive sleep apnoea
Sleep apnoea

ICD-10 term
Sleep apnoea

eTable 11 – Codes grouped as Visual impairment

CTV3 Terms
Better eye: low vision, Lesser eye: profound VI
Blind left eye
Impaired vision
Low vision, one eye
Mild visual impairment
Partial sight
Visual loss NOS
Visual loss, both eyes unqualified
ICD-10 term
Blindness - both eyes
Blindness - one eye
Blindness, binocular
Blindness, both eyes
Severe visual impairment, binocular
Severe visual impairment, monocular
Unspecified visual impairment (binocular)
Unspecified visual loss
Visual field defects

eTable 12 – Codes grouped as Hearing impairment

CTV3 Terms
Bilateral congenital sensorineural hearing loss
Bilateral deafness
Congenital sensorineural deafness
Deafness symptom
Low frequency deafness
Mix cond/sensneurl hear loss,unlat unrestrc hear/contrlat sd
Mixed conductive and sensorineural hearing loss
Mixed conductive and sensorineural hearing loss, bilateral
Moderate sensorineural hearing loss
Neural hearing loss
Profound sensorineural hearing loss
Sensorineural hearing loss
Sensorineural hearing loss, bilateral
Severe sensorineural hearing loss
Unilateral deafness
ICD-10 term
Mixed conductive & sensorineural hearing loss - bilateral

Mixed conductive and sensorineural hearing loss, bilateral
Mixed conductive and sensorineural hearing loss, unilateral with unrestricted hearing on the contralateral side
Sensneurl hear loss unilat unrestrict hear contralat side
Sensorineural hearing loss - bilateral
Sensorineural hearing loss - unspecified
Sensorineural hearing loss, bilateral
Sensorineural hearing loss, unilateral with unrestricted hearing on the contralateral side
Sensorineural hearing loss, unspecified

eTable 13 – Codes grouped as Developmental delay (also known as 'early developmental impairment")

CTV3 Terms
Developmental delay
Global developmental delay
Delayed milestone
Development delay NOS
Specific delays in development
Other development delays
Toileting problems
Not yet toilet trained
ICD-10 term
Lack of expected normal physiologic development unspec
Lack of expected normal physiological development, unspecified
Delayed milestone
Nonorganic enuresis
Nonorganic encopresis

eTable 14 – Codes unclassified / 'general' developmental disorder

CTV3 Terms
DLA 370 Disability living allowance completed
Developmental disorder
Developmental Problem
Developmental disorder NOS
Developmental dysfluency
Disability NOS
Disorder of psychological development
Mixed disorder of psychological development
Registered disabled
ICD-10 term
Unspecified disorder of psychological development
Mixed specific developmental disorders
Other disorders of psychological development

Class enumeration

Ten repetitions were done, with different starting values. [7] Higher maximum iterations of 3,000 and 8,000 were set for the 4 and 5 class models respectively. [8]

eTable 15 - Model fit indices for Latent Class analysis models with 1 to 5 classes. LL = log-likelihood; AIC = Akaike information criterion; BIC = Bayesian information criterion; cAIC = consistent Akaike information criterion; saBIC = sample-size adjusted BIC

Class	LL	likelihood-ratio	AIC	BIC	cAIC	saBIC	relative entropy
1	-13177.8	5540.1	26383.7	26488.5	26502.5	26444.0	NA
2	-11067.7	1319.7	22193.3	22410.4	22439.4	22318.3	0.939
3	-10887.6	959.5	21863.1	22192.5	22236.5	22052.7	0.953
4	-10815.0	814.4	21748.0	22189.7	22248.7	22002.2	0.953
5	-10788.2	760.3	21723.9	22278.4	22352.4	22043.2	0.931

eFigure 3 - Elbow plot of AIC = Akaike information criterion; BIC = Bayesian information criterion; cAIC = consistent Akaike information criterion; SABIC = sample-size adjusted BIC

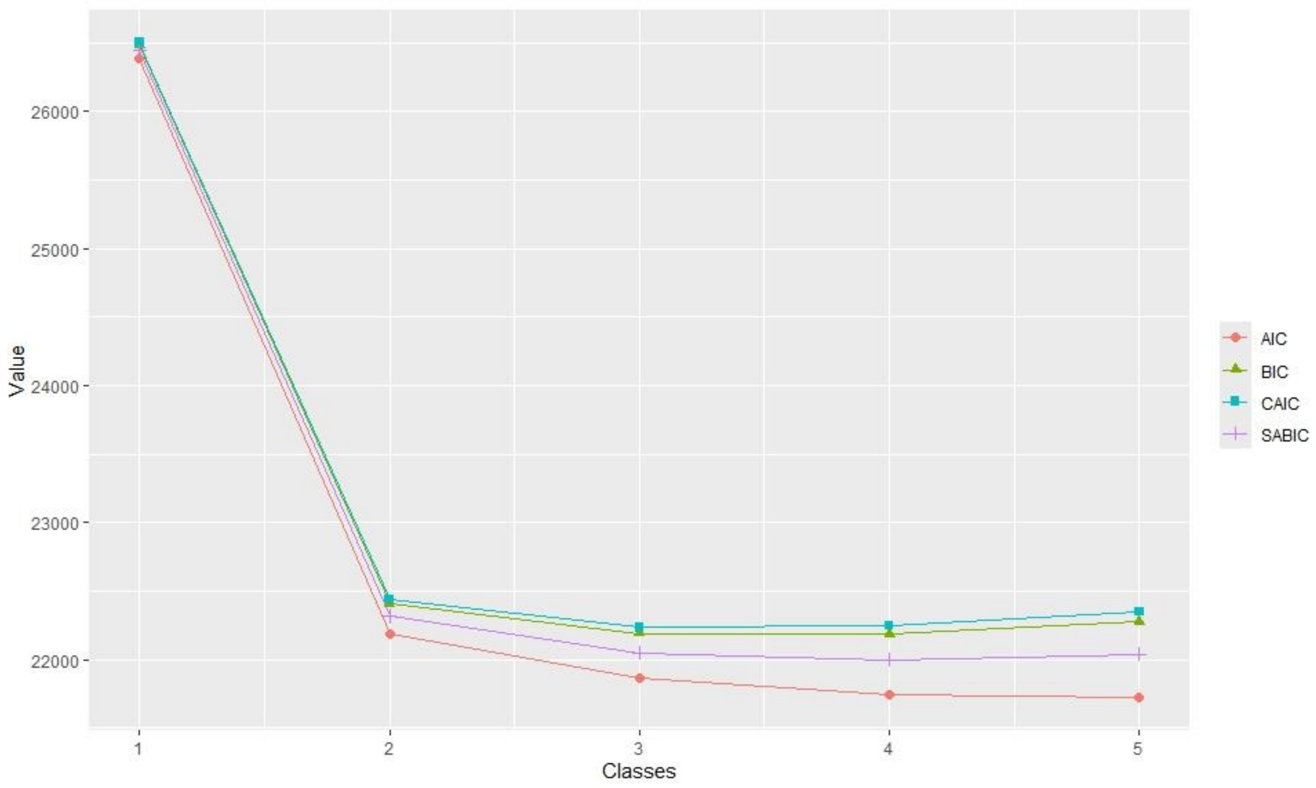

eFigure 4 – Distributions of predicted probabilities for each participant across four latent classes, divided by assigned and non-assigned groups

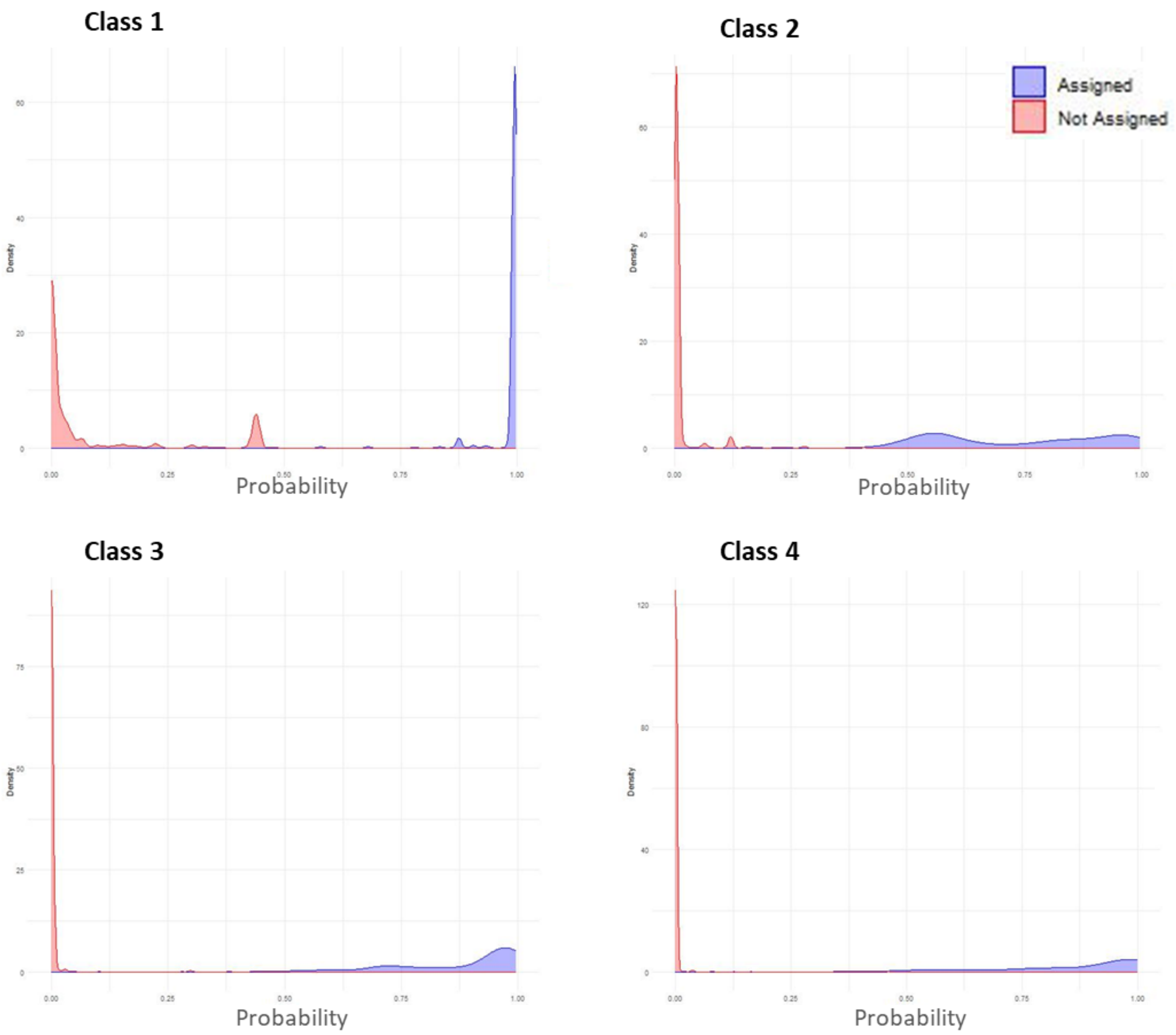

Missing data

The co-variables contained the following proportions of missing data: child's sex (0% - complete), SGA (2.4%), maternal age at delivery (0%), maternal smoking in pregnancy (17.4%), socio-economic position (17.7%), child's ethnicity (0.6%).

eTable 16 – Imputation diagnostics. Proportion of children in each category according by observed, imputed, and completed

	Observed	Imputed	Completed
**Small for gestational age (SGA)**			
n	12,862	310	13,172
not SGA	0.86	0.91	0.862
SGA	0.14	0.09	0.138
**Smoking**			
n	10,885	2,287	13,172
no smoking	0.836	0.842	0.837
smoking	0.164	0.158	0.163
**Socio-economic position**			
n	10,843	2,329	13,172
Least deprived and most educated	0.193	0.178	0.191
Employed, not materially deprived	0.199	0.186	0.197
Employed, no access to money	0.153	0.154	0.153
Benefits not materially deprived	0.295	0.31	0.298
Most economically deprived	0.16	0.173	0.162
**Ethnicity**			
n	13,099	73	13,172
Asian	0.532	0.562	0.532
White	0.382	0.384	0.382
Mixed	0.054	0.055	0.054
Black	0.019	0	0.019
Other	0.014	0	0.014

Twenty five imputations were carried out. Pre-eclampsia, gestational diabetes, consanguinity, and maternal parity were included as auxiliary variables. The largest relative variance increase was 0.05, the largest fraction of missing information was 0.26.

eTable 17 – Cohort description. Comparison of whole Born in Bradford cohort and, analysis cohort. Values are number (column percentage) unless stated otherwise. For the analyses children born <34 weeks were combined into one group due to small numbers.

**Characteristics**	**Whole cohort (n = 13,858)**		**Analysis Cohort (n = 13,172)**	
	Number	percentage	Number	percentage
Gestational age				
<28 weeks	32	0.2%	21	0.2%
28 - 31 weeks	118	0.9%	99	0.8%
32 - 33 weeks	110	0.8%	94	0.7%
34 - 36 weeks	646	4.8%	606	4.6%
37 - 38 weeks	3,028	22.4%	2,950	22.4%
39 - 41 weeks	9,407	69.5%	9,222	70.0%
>41 weeks	185	1.4%	180	1.4%
Missing	332		excluded	
Sex				
Male	6,972	51.6%	6,801	51.6%
Female	6,553	48.5%	6,371	48.4%
Missing	333		nil	
Multiplicity				
Singletons	13,199	97.6%	12,867	97.7%
Twins	318	2.4%	305	2.3%
Higher order	9	0.1%	excluded	
Missing	332		nil	
Small for gestational age				
Not SGA	11,337	85.9%	11,067	86.0%
SGA	1,857	14.1%	1,795	14.0%
Missing	664		310	
Maternal age at delivery				
Median (range)<br>Mean (SD)	27 (15-49)<br>27.5 (5.6)		27 (15-49)<br>27.5 (5.6)	
Missing	0		0	
Maternal smoking in pregnancy				
Yes, any	1,885	16.5%	1,782	16.4%
No	9,568	83.5%	9,103	83.6%
Missing	2,405		2,287	
Socio-economic position				
Least deprived, most educated	2,240	19.6%	2,096	19.3%
Employed not materially deprived	2,273	19.9%	2,160	19.9%
Employed no access to money	1,735	15.2%	1,656	15.3%
Benefits but coping	3,347	29.4%	3,201	29.5%
Most deprived	1,809	15.9%	1,730	16.0%
Missing	2,454		2,329	
Ethnicity				
Asian	7,249	52.7%	6,970	53.2%
Mixed	737	5.4%	704	5.4%
White	5,300	38.6%	5,001	38.2%
Black	270	2.0%	245	1.9%
Other	191	1.4%	179	1.4%
Missing	111		73	

References

1. Carter B, Verity Bennett C, Bethel J*, et al.* Identifying cerebral palsy from routinely-collected data in England and Wales. *Clin Epidemiol*. 2019;**11**:457-68 doi: doi:10.2147/clep.S200748 [published Online First: 20190605].

2. Swann OV, Lone NI, Harrison EM*, et al.* Studying the Long-term Impact of COVID-19 in Kids (SLICK). Healthcare use and costs in children and young people following community-acquired SARS-CoV-2 infection: protocol for an observational study using linked primary and secondary routinely collected. *BMJ Open*. 2022;**12**:e063271 doi: doi:10.1136/bmjopen-2022-063271 [published Online.

3. Uysal S. ICD-10-CM Diagnosis Coding for Neuropsychological Assessment. *Archives of Clinical Neuropsychology*. 2018;**34**:721-30 doi: doi:10.1093/arclin/acy084 [published Online.

4. Kuan V, Denaxas S, Gonzalez-Izquierdo A*, et al.* A chronological map of 308 physical and mental health conditions from 4 million individuals in the English National Health Service. *The Lancet Digital Health*. 2019;**1**:e63-e77 doi: doi:10.1016/S2589-7500(19)30012-3 [published Online.

5. Bennett Institute for Applied Data Science. OpenCodelists. 2022.

6. NHSDigital. Classifications Browser ICD-10-5TH-Edition. 2011.

7. Nylund-Gibson K, Choi AY. Ten frequently asked questions about latent class analysis. *Translational Issues in Psychological Science*. 2018;**4**:440-61 doi: doi:10.1037/tps0000176 [published Online.

8. Linzer DA, Lewis JB. poLCA: An R Package for Polytomous Variable Latent Class Analysis. *Journal of Statistical Software*. 2011;**42**:1 - 29 doi: doi:10.18637/jss.v042.i10 [published Online.
